# A retrospective survey study of paramedic students’ exposure to SARS-CoV-2, participation in the COVID-19 pandemic response, and health-related quality of life

**DOI:** 10.1186/s13049-021-00967-2

**Published:** 2021-10-18

**Authors:** Kristin Häikiö, Jeanette V. Andersen, Morten Bakkerud, Carl R. Christiansen, Kim Rand, Trine Staff

**Affiliations:** 1 Bachelor Program in Paramedic Science, Institute of Nursing and Health Promotion, Faculty of Health Science, Oslo Metropolitan University, St. Olavs plass, P.O. Box 4, 0130 Oslo, Norway; 2 HØKH – Health Services Research Unit, Akershus University Hospital, P.O. Box 1000, 1478 Lørenskog, Norway

**Keywords:** Emergency medical services, Students, Pandemics, COVID-19, SARS-CoV-2, Quality of life, Health personnel, Emergency medical technicians, Ambulances

## Abstract

**Background:**

Healthcare workers have reported increased anxiety while working in hospitals during the COVID-19 pandemic, and the role of healthcare students in a health crisis has been discussed among clinicians and researchers. The simultaneous international shortage of personal protection equipment (PPE) during the first wave of the pandemic potentially exposed healthcare workers and students to the virus during their work and clinical training. Our aim was therefore to evaluate the extent to which paramedic students in Oslo, Norway, were exposed to the SARS-CoV-2 virus and were involved in voluntary and/or paid healthcare-related work. An evaluation was also made of the students’ COVID-19-related symptoms and of their health-related quality of life (HRQoL) during the first wave of the pandemic.

**Methods:**

Paramedic students (n = 155) at Oslo Metropolitan University, Norway, were invited to complete an online survey five months after the first cases of COVID-19 were detected. The university was situated in the epicenter of the pandemic in Norway. The responses were analyzed using descriptive statistics, independent sample t-tests, and linear regression analysis.

**Results:**

Of the 109 respondents (70.3%), 40 worked in patient-related healthcare work. Of those, seven (17.5%) students experienced insufficient supplies of PPE, six (15.0%) participated in aerosol-generating procedures without adequate PPE, and nine (22.5%) experienced insufficient time to don PPE. Seventy-five (70.1%) students experienced no COVID-19-related symptoms, and no students tested positive for COVID-19. HRQoL was scored 0.92 (sd 0.12), which was significantly higher than for the general population before the pandemic (*p* = 0.002). Students continued with their education and participated in a variety of pandemic-related emergency tasks during the first wave of the pandemic.

**Conclusions:**

Paramedic students were valuable contributors to the national pandemic response. Despite potential exposure to SARS-CoV-2 in unpredictable emergency settings with limited supplies of personal protection equipment, no students tested positive for COVID-19. Their health-related quality of life remained high. Students’ participation and utilization in similar health crises should be considered in future health crises.

## Introduction

The severe acute respiratory syndrome coronavirus 2 (SARS-CoV-2), causing the disease known as Coronavirus disease 2019 (COVID-19), rapidly spread worldwide in the first months of 2020, and was declared a global pandemic by the World Health Organization (WHO) on March 11, 2020. At this time, Norway had the seventh-highest confirmed rates of infection worldwide, at 110 cases per million inhabitants [1]. Paramedics and other healthcare workers were exposed to the new virus with limited knowledge about its degree of infectiousness, mode of transmission, or virulence. Simultaneously, there were national [2, 3] and international [4] shortages of personal protection equipment (PPE), which constituted a risk for healthcare workers of catching COVID-19 while performing their work [3]. This made healthcare work challenging and unpredictable.

Recent results have demonstrated that ambulance staff in Norway had one of the highest COVID-19 infection incidence rates (1.83%) during 2020 compared with other healthcare groups (1.48% for all healthcare employees) [5]. Ambulances are often assigned to situations where the patient’s background and the emergency situation, such as infection with COVID-19, were unclear [6]. For example, patients with COVID-19 may present with critical hypoxemia without proportional signs of respiratory distress [7].

Several studies of COVID-19 patients with respiratory failure acknowledge that paramedics in a prehospital setting are a valuable resource for evaluating and treating COVID-19 patients [8, 9], Consequently, paramedics are valuable healthcare workers during the COVID-19 pandemic, but they need to take precautions and use PPE whenever they suspect COVID-19 in patients, often in a time-critical phase.

Previous studies among healthcare workers exposed to SARS-CoV-2 found that working in hospitals led to considerable levels of depression, anxiety, insomnia, distress, and even suicide [10, 11]. Lack of confidence in infection control measures may prevent adaptive responses to stress for healthcare workers [12]. Although many studies found that anxiety and distress were common among healthcare workers, there is a paucity of studies considering paramedic students’ perceptions of being part of the primary response to the COVID-19 pandemic. One study from Australia found that paramedic students experienced some level of anxiety, and that anxiety was mediated by changed approaches to studies, financial circumstances, social support, university adaptation, acceptance, and career pathway choices [13].

Following the shortage of healthcare workers in countries and regions that were hit hard early in the pandemic, such as northern Italy, discussions have taken place in several countries as to whether healthcare students, such as paramedics and nursing students, should take part in clinical work during times of crisis [14, 15]. A large study from Canada demonstrated that 77.7% of university nursing students were willing to volunteer and work during a pandemic [16], and in Australia paramedic students expressed a willingness to participate in front-line clinical work during the COVID-19 pandemic [17]. Still, there is a scarcity of literature evaluating both willingness and involvement among paramedic students’ in public health emergencies [18].

In Australia, paramedic students were defined as non-essential staff, resulting in many students being faced with postponed clinical placements and delayed graduation [17]. During the first wave of the pandemic (spring of 2020) in Norway, the Norwegian government announced that all healthcare students could be enrolled in the national COVID-19 response plans to increase the number of healthcare workers available [19]. This decision also included paramedic students although several studies have focused on the risks of paramedics acquiring infectious diseases [6, 20],

Paramedic prehospital work is often performed in chaotic and complex settings and during a pandemic even under threat to personal safety. Still, the governments need to increase the number of available health-care personnel workers, depends heavily on healthcare students` contribution. A potential consequence of our study could be to increase the local and national authorities´ attention towards paramedic students role in local and national response plans and to highlight the potential benefits for the health-care response.

## Method

### Study design

This was a retrospective, descriptive, cross-sectional study in which all bachelor-level paramedic students (n = 155) enrolled at Oslo Metropolitan University (OsloMet) were invited to complete an electronic survey in June 2020, five months after the initial outbreak of COVID-19 in Norway in February 2020.

### The context

The three-year bachelor program in paramedic science at OsloMet is the largest of four such programs in Norway, with 158 students enrolled in the spring of 2020. The program consists of theoretical training, simulation training, and training in study-related clinical placements. In study-related clinical placements, the students were primarily divided between the prehospital services in the South-Eastern Norway Regional Health Authority. The services consisted of 103 ambulance stations with a total of 214 regular ambulances [21].

Most of these students had their study-related clinical placements in the ambulance department at Oslo University Hospital (OUH), meaning they were working at the epicenter of the pandemic. The ambulance department at OUH covered a population of 697,010 inhabitants [22] and consisted of a total of 450 paramedics and emergency medical technicians who carried out 150,000 missions per year [23]. Due to the lack of PPE available in the clinical, half of the second-year class had to reshuffle their study plan so that their study-related clinical placements were postponed for three months. Consequently, 24 out of 48 second-year paramedic students were in study-related clinical placements in OUH (Bjelde, T. Private communication, April 2, 2020).

Because of the shortage of ambulance staff due to high rates of sick leave and quarantine, some paramedic students who attended study-related clinical placements were required to work unsupervised as part of the ambulance service’s professional staff instead of being supervised. The unsupervised clinical placement was accredited their study progression as mandatory study-related clinical placements. This was based on a special exemption for national practice placements sanctioned by the Ministry of Education and Research due to the pandemic [19]. Of the 24 second-year students in study-related clinical placements, 18 students were supervised while six (three second- year and three third-year students) were unsupervised (Bjelde, T. Private communication, April 2, 2020). The remaining 23  second-year students had their study-related clinical placements postponed until the fall of 2020 (Häikö, K. Private communication, June 15, 2021).

In cooperation between the bachelor program in paramedic science and the prehospital services in the South-Eastern Norway Regional Health Authority, it was decided that only students who had acquired theoretical knowledge and implemented simulation skills training in emergency medicine and in trauma and disaster management were allowed to perform paid patient-related healthcare work in the ambulances. In addition, they had to complete at least one module of study-related clinical placement. Consequently, for students to be enrolled as members of the paid working staff in the ambulance service, the requirements were: (1) completion of the first year of the bachelor program in paramedic science, (2) completion of a mandatory skill test in the respective ambulance services to prove their capability, and (3) in hold of a driver’s license. The mandatory skill tests were the same as for every other healthcare worker who seeks employment in the ambulance service. These steps were taken as a necessary precaution to ensure patient safety.

The definition and education of paramedics vary between countries [24, 25], and in this paper we apply the term “paramedic students” only to bachelor students at university level. The program is regulated by the Regulations concerning national guidelines for paramedic education. The regulations state that the aim of the bachelor program in paramedic science is to educate candidates who can promote, maintain and re-establish health and life quality for humans at individual, group, and societal level [26].

### Data collection

An electronic survey tool developed by the University of Oslo for collecting sensitive data, *Nettskjema*, was used for data collection. An invitation to participate in the study, together with an information sheet and a link to the self-administered electronic survey, was sent to students’ university email addresses in early June 2020. Reminders were sent to all students repeatedly over a three-week period, and university teachers simultaneously encouraged students to complete the survey. A promotional video of the study was made and presented to students to remind and encourage them to participate.

### Data variables

The collected variables were chosen based on previous research on how the COVID-19 pandemic had affected healthcare workers, such as levels of anxiety, depression and distress [10], and research related to virus exposure in emergency settings [6, 27].

#### Characteristics of participants

Data on gender (male, female, other) and age in years were collected.

#### Exposure to COVID-19

To measure students’ exposure to SARS-CoV-2, we asked participants about their use of PPE, COVID-19 symptoms, and COVID-19 tests. Variables regarding PPE included whether students had experienced insufficient supplies of PPE (yes/no), inadequate use of PPE during aerosol generating procedures (AGPs) (yes/no), and whether they experienced insufficient time to don PPE (yes/no).

Variables of COVID-19 symptoms included typical symptoms of COVID-19 as listed by the WHO [28] and the Centers for Disease Control and Prevention (CDC) [29] on May 22, 2020. The symptoms included fever, chills, coughing, shortness of breath, fatigue, body and muscle ache, sore throat, diarrhea, eye infection, headache, skin rashes or discoloration of fingers and toes, and a category for other symptoms. Students indicated whether they had any of these symptoms during the first three months of the pandemic (yes/no). Because it is unclear from the data whether these symptoms occurred simultaneously or not, we focus our report on the proportion of students that did not experience any COVID-19-related symptoms during the first five months after the outbreak of COVID-19 in Norway.

#### Student self-reported health-related quality of life

The students’ HRQoL was measured using the well-known and validated Euroqol’s EQ-5D-5L instrument in Norwegian translation [30]. The instrument has two parts: a health profile and a visual analog scale (EQvas). The health profile comprises five dimensions of health: mobility, self-care, usual activities, pain and discomfort, and anxiety and depression. Participants rate each dimension on a five-point scale, each point indicating the level of problems experienced (1 = *none*, 2 = *slight*, 3 = *moderate*, 4 = *severe*, 5 = *extreme/unable*) [31].

There are 3,125 possible combinations of responses (i.e., EQ-5D health states) and each response is assigned a value reflecting the population preference for the various health states (EQvalue). This value represents the preferences of the general population, and is presented on a scale where 1 denotes full health and 0 denotes a health state in which the general population on average would state indifference if asked to choose between 10 years of life and immediate death [32, 33].

In the absence of a Norwegian value algorithm for the EQ-5D, we used the EQ-5D-5L crosswalk value set for the UK [34]. The second part of EQ-5D-5L, the visual analog scale (EQvas), is a vertical scale ranging from the endpoints “worst health you can imagine” (= 0) to “best health you can imagine” (= 100) [35].

Reported HRQoL was compared to Norwegian general population norms for EQ-5D-5L for the same age/sex groups [36].

#### Students’ participation in the national response to the COVID-19 pandemic

Descriptions of students’ participation in the national pandemic response included variables grouped as 1) patient-related healthcare work and 2) non-patient-related healthcare work. The variable *patient-related healthcare work* (yes/no) identified students enrolled in 12 weeks of study-related clinical placements in an ambulance service and students who had other healthcare-related work involving patient contact (such as working in test stations, hospital wards or other healthcare institutions). The variable *non-patient-related healthcare work* (yes/no) described students who worked at COVID-19 call centers (public telephone service answering questions and giving advice regarding COVID-19 symptoms, testing procedures, quarantine, etc.); logistical work in the ambulance service, hospitals or other places; work at ambulance decontamination stations; and participation in other non-patient pandemic-related activities.

### Statistical analysis

For most analyses we used the computer software platform SPSS, version 26. KH conducted the analysis and AKH checked the data files, SPSS syntax, and outputs for errors. No errors were identified. KR conducted linear regression modelling in the computer program R, version 4.0.4 to compare the results of HRQoL to the Norwegian population.

Descriptive statistics were performed to describe participant characteristics, exposure to COVID-19, HRQoL, and participation in the national response to COVID-19. Results for categorical variables are presented with the number of cases, percentage of the sample, and missing values. Age is reported in terms of mean and standard deviation; minimum and maximum values are not reported due to the risk of identifying individual participants.

Differences in HRQoL between groups were analyzed using independent samples t-tests. Because the number of cases in some groups was small, we conducted sensitivity analyses using the non-parametric Mann Whitney U test. To compare the EQ-5D-5L responses of paramedic students with the Norwegian general population, we made use of data from a general population survey conducted in 2019 and designed to generate population norms for the instrument [36]. Considering the limited age range of the 109 paramedic students, we limited the general population data to the 1,553 respondents aged between 18 and 49 years. EQvalue and EQvas scores from the general population and paramedic student respondents were compared using linear regression modelling, controlling for age (categories for 18–29, 30–39, and 40–49 years), sex, educational level (categories for primary education, secondary education, less than four years of higher education, and at least four years of higher education), and paramedic students’ study progression in years (student seniority). With a base case corresponding to a female general population of respondents aged 18–29 with less than four years of higher education, we used two regression models for each of EQvalue and EQvas: one in which a single parameter indicated paramedic student status, and one in which paramedic students were identified by year of study. Details regarding the setup of these models can be found in “Appendix 1”.

## Ethics

The Regional Committees for Medical and Health Research Ethics approved this study (reference no. 142135). A digital information letter was provided to the students before data collection. The letter included information regarding anonymity, voluntary participation, secure data processing, and the ability to withdraw without having to give a reason. It was highlighted that even though no directly identifiable data were collected, the possibility of indirect identification existed. The latter was due to a small sample size and to the researchers’ knowledge of the students. The same information was provided verbally by faculty. In the survey, students had to actively confirm they had read and understood the information provided and were informed that responding to the survey was considered as consent to participate.

Data storage and analysis were performed using Tjenester for Sensitive Data (TSD), a specialized service for safe data processing, and data collection was performed electronically with Nettskjema. Both Nettskjema and TSD are services provided by the University of Oslo´s University Centre for Information Technology (USIT). Both are in accordance with the Norwegian Personal Data Act and the Norwegian Health Research Act. The data harvesting through Nettskjema has a direct and encrypted link to TSD for data storage, and the data management plan was approved by the Norwegian Centre for Research Data (reference no. 409603). Data deletion will be performed upon completion of the project, or by May 14, 2028 at the latest.

## Results

Of the 156 students enrolled in the bachelor program in paramedic science at OsloMet, 109 students participated in this study, which constitutes a response rate of 70.3%. The mean age of the participants was 24.6 years (sd 4.2) (Table 1). Students represented all three study years: 95.5% (42 of 44 students) of first-year students, 83% (44 of 53 students) of second-year students, and 39% (23 of 59 students) of third-year students.

**Table 1:** Characteristics of the sample (N = 109)

	Year 1	Year 2	Year 3	Total
Female, n (%)	22 (52.4)	27 (61.4)	13 (56.5)	62 (56.9)
Age in years, mean (sd)	23.3 (3.2)	25.5 (5.3)	25 (3.2)	24.6 (4.2)
Students performing patient-related healthcare work, n (%)*	2 (4.8)	23 (52.3)	15 (65.2)	40 (36.7)
Students performing study-related clinical placement, n (%)	–	20 (45.5)	–	20 (18.3)

^*^Feb-Jun 2020

Slightly more than half of the participating students were female, 40 students performed patient-related healthcare work during the spring 2020, and 20 students were in study-related clinical placements in an ambulance service (Table 1). All 20 participating students who were in study-related clinical placements were in their second year.

### Exposure to COVID-19

#### To what extent did students in patient-related healthcare work experience lack of PPE?

Among the 40 students who performed patient-related healthcare work, seven students (17.5%) reported experiencing insufficient supplies of PPE available, and six students (15.0%) reported having participated in AGPs without adequate PPE. Nine students (22.5%) reported experiences of not having enough time to don PPE.

Of the 20 students who were in study-related clinical placements in an ambulance service between March and June 2020, one student (5%) reported having experienced insufficient supplies of PPE and three students (15%) participated in AGPs without adequate PPE. Five students (25%) experienced having no time to don PPE.

### Symptoms related to COVID-19

Most the 109 students participating in this study (n = 75, 70.1%) experienced no symptoms related to COVID-19 during the study period. Of those who did report COVID-19-related symptoms (n = 39, 29.9%), the most common were: sore throat, n = 26 (26%), coughing n = 15 (14%), and headache n = 11 (10.3%) (Table 2).

**Table 2:** Distribution of students with no symptoms, and students who tested for COVID-19 s (N = 109)

	1st-year students		2nd-year students		3rd-year students		All students	
No symptoms, n (%)	27.0	(65.9)	33.0	(75.0)	15.0	(68.2)	75.0	(70.1)
Tested for COVID-19, n (%)	2.0	(4.8)	4.0	(9.1)	5.0	(21.7)	11.0	(10.1)

Of all 109 students, only 10.1% (n = 11) were tested for COVID-19 (Table 2). No students tested positive for COVID-19. Of the 34 students who reported having had COVID-19-related symptoms, 11 were tested, and none tested positive.

### How was students’ health-related quality of life five months after the outbreak of COVID-19?

#### Descriptions of paramedic students’ health-related quality of life

Students mean HRQoL was 0.92 (sd 0.12) when measured with EQvalue, and 82.9 (sd 12.0) when measured with EQvas (Table 3).

**Table 3:** Health-related quality of life and the EQ-5D-5L domains across study years, N=109

	First-year students			Second-year students			Third- year students		
	Mean	SD	Missing	Mean	SD	Missing	Mean	SD	Missing
**Health-related quality of life**									
EQvas	79.7	(12.8)	0	83.1	(11.4)	0	88.4	(9.8)	0
EQvalue	0.9	(0.15)	0	0.92	(0.099)	0	0.96	(0.07)	1

**EQ-5D-5L dimensions**	*n*	%		*n*	%		*n*	%	
*MOBILITY*									
No problem	40	(90.9)	0	43	(97.7)	0	21	(95.5)	1
Slight problem	2	(4.5)		1	(2.3)		1	(4.5)	
*SELFCARE*									
No problem	42	(100)	0	42	(100)	0	23	(100)	0
Slight problems	0	(0)		1	(2.4)		0	(0)	
Moderate problems	0	(0)		1	(2.4)		0	(0)	
*USUAL ACTIVITES*									
No problem	31	(73.8)	0	37	(84.1)	0	33	(78.6)	0
Slight problem	7	(16.7)		4	(9.1)		7	(30.4)	
Moderate Problems	3	(7.1)		2	(4.5)		1	(4.3)	
Severe problems	1	(2.4)		1	(2.3)		1	(4.3)	
*PAIN OR DISCOMFORT*									
No problem	33	(78.6)	0	39	(88.6)	0	21	(91.3)	0
Slight problems	7	(16.7)		5	(11.4)		2	(8.7)	
Moderate Problems	1	(2.4)		0	(0.0)		0	(0.0)	
Extreme problems	1	(2.4)		0	(0.0)		0	(0.0)	
*ANXIETY OR DEPRESSION*									
No problem	28	(66.7)	0	28	(63.6)	0	19	(82.6)	0
Slight problems	13	(31.0)		14	(31.8)		4	(17.4)	
Moderateproblems	1	(2.4)		2	(4.5)		0	(0.0)	
Extreme problems	0	(0.0)		0	(0.0)		0	(0.0)	

There were differences between the student cohorts, indicating that HRQoL was significantly higher among third-year students than among the other students (EQvalue: *p* = 0.000 CI − 0.08, 0.02; EQvas: *p* = 0.000 CI − 9.18, 2.26).

#### No differences in the anxiety/depression domain between students who did or did not experience insufficient supplies of or inadequate PPE

There were no significant differences in students’ anxiety/depression between group 1 (those who experienced insufficient supplies of PPE) and group 2 (those who did not experience insufficient supplies of PPE) (*p* = 0.87 CI − 0.29, 0.35). Nor were there any significant differences in students’ HRQoL measured with Eqvalue (*p* = 0.97 CI − 0.05, 0.05) or Eqvas (*p* = 0.17 CI − 11.19, 2.04) between the two groups.

Furthermore, no significant differences were found between the groups who experienced and did not experience inadequate PPE during AGPs when it came to anxiety/depression (*p* = 0.09 CI − 0.71, 0.53), or HRQoL measured with Eqvalue (*p* = 0.27 CI − 0.03, 0.13) or Eqvas (*p* = 0.92 CI:-8.74, 7.86).

A sensitivity analysis confirmed the above results.

#### Paramedic students’ HRQoL compared with the Norwegian general population

**Fig. 1:**
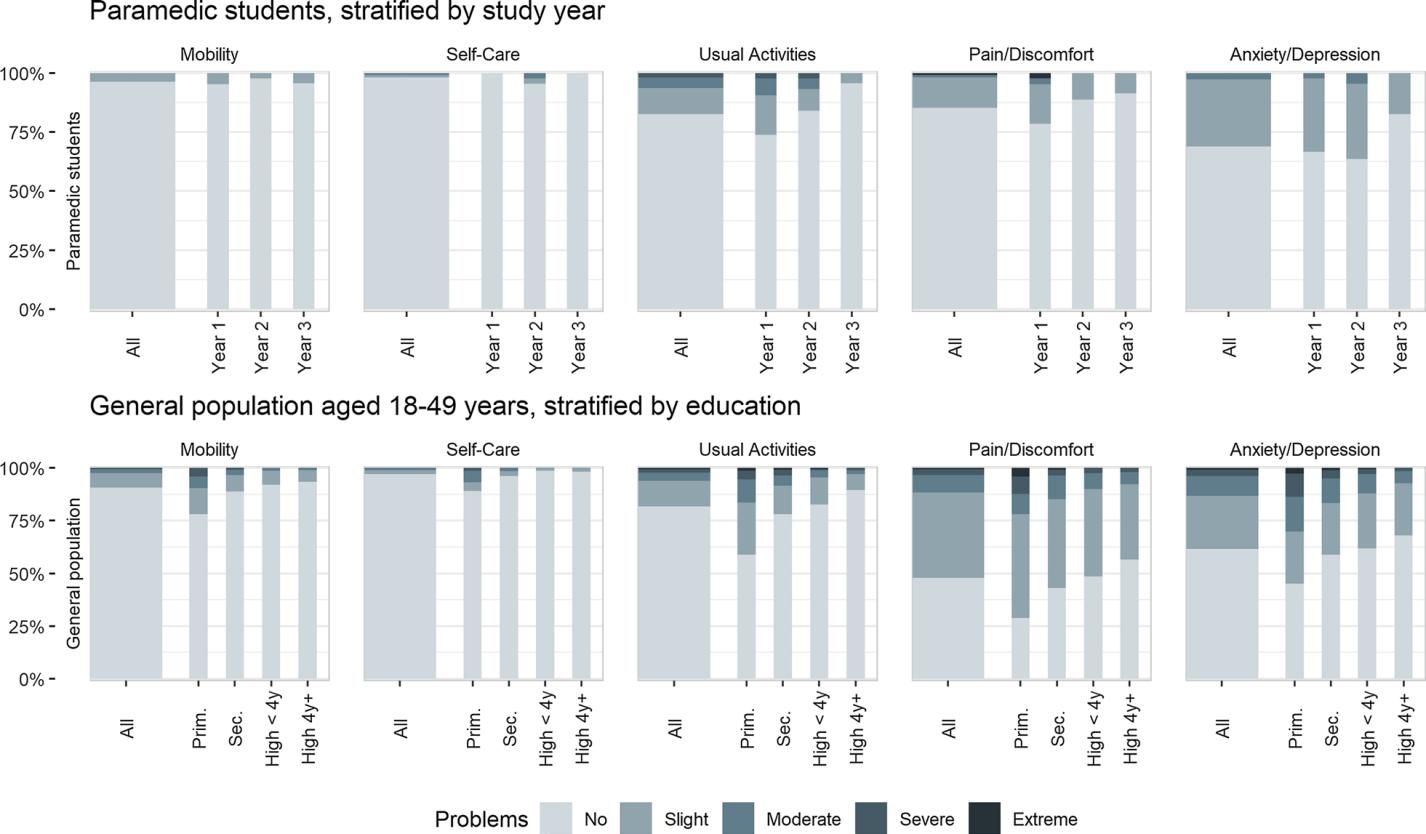
Paramedic students’ response to each dimension of the EQ-5D descriptive system compared with the general population in Norway

Paramedic students indicated better HRQoL five months into the pandemic than did the general population before the pandemic, particularly in the pain/discomfort and the anxiety/depression domains (Fig. 1).

Controlling for age, educational level of the general population sample, and sex, the regression models indicate that paramedic students reported statistically significant fewer health problems on the EQ-5D-5L than the general population sample from 2019, with a mean difference in EQvalue of 0.057 (*p* < 0.001). In terms of EQvas, there was a statistically non-significant difference of 0.811 (*p* = 0.636). There was a trend towards higher reported EQvalue and EQvas with increasing student seniority (Table 4).

**Table 4:** Regression models comparing paramedic respondents to general population respondents aged 18–49 years

	EQvalue (Utilities)						EQvas					
	All paramedics			By year			All paramedics			By year		
Predictor	Est	(SE)	*p*	Est	(SE)	*p*	Est	(SE)	*p*	Est	(SE)	*p*
Intercept *	0.849	(0.01)	< 0.001	0.849	(0.01)	< 0.001	81.473	(0.924)	< 0.001	81.374	(0.923)	< 0.001
Age												
Age 18–29*		–			–			–			–	
Age 30–39	0.003	(0.011)	0.789	0.003	(0.011)	0.806	0.401	(1.006)	0.691	0.403	(1.006)	0.689
Age 40–49	− 0.036	(0.011)	< 0.001	− 0.036	(0.011)	< 0.001	− 3.196	(1.026)	0.002	− 3.167	(1.025)	0.002
**Sex**												
Female *		–			–			–			–	
Male	0.032	(0.009)	< 0.001	0.032	(0.009)	< 0.001	1.371	(0.828)	0.098	1.414	(0.828)	0.088
**Education**												
Primary education	− 0.141	(0.021)	< 0.001	− 0.141	(0.021)	< 0.001	− 11.45	(1.984)	< 0.001	− 11.50	(1.982)	< 0.001
Secondary education	− 0.038	(0.011)	< 0.001	− 0.038	(0.011)	< 0.001	− 2.950	(1.046)	0.005	− 2.874	(1.045)	0.006
Higher, short *		–			–			–			–	
Higher, long	0.030	(0.012)	0.009	0.030	(0.012)	0.009	1.905	(1.093)	0.082	1.986	(1.092)	0.069
**Group**												
General population *		–			–			–			–	
Paramedic	0.057	(0.018)	0.002		–		0.811	(1.711)	0.636		–	
Year 1		–		0.035	(0.027)	0.198		–		− 2.314	(2.533)	0.361
Year 2		–		0.061	(0.026)	0.022		–		1.279	(2.463)	0.604
Year 3		–		0.091	(0.036)	0.012		–		6.856	(3.319)	0.039

*Base case: Female general population respondent aged 18–29 with less than five years of higher education

### What kind of healthcare-related work did the students perform during the first five months of the pandemic?

Students took part in various pandemic-related activities and contributed to the national COVID-19 response. The results are presented below.

#### Students’ participation in the national COVID-19 response through patient-related healthcare work

Of all 109 participants, n = 40 (36.7%) performed patient-related healthcare work during the pandemic (Table 4). |Of these 40 students, n = 20 (50%) were included in study-related clinical placements in an ambulance service. The other 20 students performed other patient-related healthcare work, such as paid work in other healthcare institutions. Two of the 40 students worked at a COVID-19 test center and performed other healthcare-related work (Table 5).

**Table 5:** Student activity in patient-related healthcare work as part of COVID-19 response (N = 109)

	n	(%)
Patient-related healthcare work	40	(36.7)
Study-related clinical placement in ambulances	20	(18.3)
Other healthcare-related work	20	(18.3)
COVID-19 test-center*	2	(1.8)

^*^These two students also performed other healthcare-related work

### Students’ participation in the national COVID-19 response through non-patient-related healthcare work

Eleven of the 109 paramedic students (10%) were assigned to ambulance decontamination stations (Table 6). These were set up due to many ambulances needing decontamination after delivering patients with suspected COVID-19. A further n = 10 (9.2%) performed logistical work for the ambulance service. The logistical work consisted of packing and distributing PPE and other medical consumables for the ambulance service.

**Table 6:** Student activity in non-patient-related healthcare work as part of the COVID-19 response (N = 109)

	n	%
Non-patient-related healthcare work	20	(18.3)
Work at ambulance decontamination station	11	(10.2)
Logistical work at hospital/ambulance service*	10	(9.2)
COVID-19 clinical information telephone service	3	(2.8)
Other activities	5	(4.6)

^*^Four of these 10 students also worked at a decontamination station, and one also worked on logistics

A few students were assigned to the COVID-19 clinical information telephone service (n = 3, 2.8%) which was set up to answer questions from members of the public regarding COVID-19. Three students performed paid work at the COVID-19 test centers. No students reported performing voluntary work for the Red Cross, the Norwegian Civil Defence or similar organizations.

## Discussion

This study was part of a larger retrospective evaluation of paramedic students’ experiences in the context of the first wave of the COVID-19 pandemic in Oslo, Norway, in spring 2020. Analyses revealed that among paramedic students who were part of the national response to COVID-19, few experienced lack of PPE, and none tested positive for COVID-19, despite performing clinical healthcare-related work during the national and international shortage of PPE [2–4].

Compared with the general Norwegian population, paramedic students reported significantly better HRQoL, and there was a trend towards higher scores on both EQvalue and EQvas with increasing student seniority despite the risks associated with ambulance work during a pandemic.

Students participated in the national pandemic response through both patient-related and non- patient-related healthcare work.

### Students’ exposure to SARS-CoV-2

Our study shows that seven (17.5%) of the 40 students who performed patient-related healthcare work experienced insufficient supplies of PPE. Among the 20 students who were in study-related clinical placements in an ambulance service, only one student (5%) experienced insufficient supplies of PPE. SARS-CoV-2 is currently known to spread from the infected persons’ nose and mouth in small liquid particles containing the virus, called aerosols or droplets, to people who inhale or who are in direct contact with the their eyes, nose, or mouth [37]. PPE is known to prevent the transmission of viruses and, consequently, of infectious diseases such as COVID-19. Previous reports have shown that there was a lack of appropriate PPE many places around the world during the first wave of COVID-19 [4]. The explanation for the low number of students experiencing insufficient supplies of PPE in our study may be that Norway prioritized 70% of available PPE to ambulance services and other specialist health services over primary care [38]. In addition, the number of students in study-related clinical placements was adjusted according to the availability of PPE in the ambulance service.

Our results show that six students (15.0%) participated in AGPs without adequate PPE while performing patient-related healthcare work, and three students (15%) participated in AGPs without adequate PPE while in study-related clinical placements in the ambulance service. COVID-19 patients may need supplemental oxygen therapy, and sometimes non-invasive or invasive ventilator support [7, 8, 39]. In Norway, ambulance personnel perform AGPs such as continuous positive airway pressure (CPAP) and invasive airway interventions such as extraglottic airway devices and endotracheal intubations. AGPs are known to constitute a risk to personnel in the proximity [8, 9, 40]. These numbers indicate that students were exposed to COVID-19 through emergency work in and outside the ambulance service. The reason for participating in AGP without sufficient PPE could be because situations occur unexpectedly and need urgent intervention from healthcare workers, and because different patient situations require different use of PPE. For example, unexpected cardiac arrest. The relatively high number of students reporting insufficient time to don PPE could indicate that the acute setting is a stronger predictor of inadequate use of PPE than availability in emergency settings.

Considering that 15% of the students experienced inadequate PPE during AGPs, it is also possible that there is a lack of adequate operating procedures for PPE among ambulance crews. The experience of not having enough time to don PPE may result from the nature of acute emergency situations, but it may also be related to lack of experience in donning PPE, lack of familiarity with new types of PPE, lack of knowledge about which type of PPE to use, and lack of training. In time-critical settings, ambulance crews will always have to assess whether to apply PPE before the ambulance leaves the station or when it arrives at the scene, especially if they are redirected from one mission to another.

Despite some suboptimal experiences of using PPE among the paramedic students, the results demonstrate that no students tested positive for COVID-19. It is worth pointing out that testing capacity at this stage of the pandemic was limited [41] and that asymptomatic students were not tested. Consequently, students may have been positive for COVID-19 without being symptomatic [42]. Moreover, contracting COVID-19 is not necessarily a result of a lack of PPE or even of work-related activities; it can just as well come from contact with family, friends, or other social relationships [5]. Finally, we must emphasize that our finding of no students testing positive for COVID-19 is only representative of the first five months of the pandemic, which was the first wave of the pandemic, and at this early stage the SARS-CoV-2 variants where less transmissible than subsequent variants [43]. Later in 2020 and 2021, the number of people in the general population that tested positive for COVID-19 rose significantly, and cases were reported of paramedic students testing positive (unpublished results).

### Health-related quality of life

#### Paramedic students reported higher health-related quality of life than the general population

Compared with the general Norwegian population measured prior to the pandemic, Norwegian paramedic students reported fewer health problems and better HRQoL, as measured by the EQ-5D-5L descriptive system, five months into the pandemic. A mean difference in EQvalue of 0.057 would typically be considered clinically significant. Taken literally, a value of 0.057 means that people would forgo 5.7% more of their remaining lifetime to avoid the problems reported by the average Norwegian general population than they would to avoid the problems reported by paramedic students. This indicates that paramedic students reported generally good health, which may reflect the general health of individuals who choose this type of education. In terms of subjective HRQoL today, as measured by the EQvas, the students were not statistically different from the general population, controlling for age, sex, and educational level.

Interestingly, we observe that both EQvas and EQvalue increase with student seniority (study year). Whether this reflects a general trend, a cohort-specific effect, or different experiences due to diverging study situations during the pandemic cannot be determined from this study alone. However, a study currently in pre-print that reports on QoL and fear of COVID-19 among Norwegian nursing students during the pandemic also reports lower levels of fear of COVID-19 in the more senior student cohorts [44]. This is interesting because fear of COVID-19 is likely to affect the EQvalue score through the anxiety/depression domain. Longitudinal follow-up would be helpful to improve interpretation.

#### The anxiety/depression domain among students and healthcare workers

The anxiety/depression domain of HRQoL was the domain where the students reported most problems. Still, their overall HRQoL measured with the descriptive part of EQ-5D-5L was significantly higher than the general population. In contrast, several studies among healthcare worker exposed to SARS-CoV-2 in hospital settings have found that healthcare workers experienced considerable levels of depression, anxiety, insomnia, distress, and even suicide [10, 11]. Anxiety and sleep disturbance among healthcare workers dealing with COVID-19 patients were also found in a study from India, especially among young female healthcare workers. This contrasts with our findings, where paramedic students had higher HRQoL that increased with student seniority. This is despite their participation in clinical patient-related work during the pandemic in their second and final years of study. It is worth mentioning that Norway fared well in handling the SARS-CoV-2 virus due to good public welfare, a strong public healthcare system and a high level of trust in the population [45]. These factors may have reduced students’ anxiety/depression compared with other countries. Although anxiety/depression is only one of the domains of the EQ-5D-5L instrument, we would have expected to see a larger impact on students’ HRQoL if anxiety/depression were dominant symptoms among the paramedic students. Most of the paramedic students in the study were young (aged 18–29), and a small majority were female, indicating that the distribution of these characteristics does not explain the low score on anxiety/depression.

#### The anxiety/depression domain in relation to lack of personal protection equipment

In our study, no significant differences in HRQoL or anxiety/depression were found between those who experienced insufficient supplies or inadequate use of PPE and those who did not. In contrast to our findings, a study among nursing students in the United States found a correlation between lack of PPE and higher anxiety [46, 47]. One reason for the differences in results between the Norwegian paramedic students and the US nursing students may be the different tools used to measure anxiety/depression. We measured anxiety/depression with one of the descriptive domains of EQ-5D-5L, which may not be as sensitive as the instrument used in the US study. However, we cannot rule out the possibility that the bachelor program in paramedic science may attract students with personality types other than those described in earlier studies. Moreover, the Norwegian study context, with the Scandinavian welfare model of tax-financed healthcare available to all inhabitants, may play a role in reducing the consequences for students’ personal life, financial circumstances and other factors if they were to contract COVID-19, and this may reduce negative stress or anxiety compared with students in the US health-system context. Finally, the relative death rate from COVID-19 in the United States was 13 times higher than in Norway [1] which is also likely to affect anxiety levels in the two countries differently.

### Were paramedic students trained and ready, but under-utilized during the first wave of the pandemic?

Unlike Australia, Norway’s Ministry for Education and Research made a formal decision to integrate healthcare students, including paramedics, in the broader national healthcare response [17–19]. We found that a total of 61 paramedic students (56%) at OsloMet participated in the national response during the first wave of the pandemic. Among these, 21 (19%) first-year student paramedics were enrolled in non-patient-related healthcare work and were a valuable resource despite their lack of clinical patient experience.

Among the 20 student paramedics in study-related clinical placements, only three second^−^year students were considered as trained, ready, and suitable to be enrolled in the national response to the pandemic for paid, non-supervised patient-related work. Recent findings demonstrate that interventions ranging from simple classroom-based interactive discussions to complex multimodal simulative experiences result in improved knowledge, skills, and attitudes towards participating in disaster medicine scenarios such as a pandemic [48]. However, our results indicate that training through study-related clinical placements seems to be a crucial factor for being evaluated and considered trained, ready and suitable.

Our results show that the three students who were considered as trained, ready and suitable by the OUH to be enrolled in the national response to the COVID-19 pandemic for paid, non-supervised patient-related healthcare work were second-year students. They had acquired both the theoretical knowledge and the skills through simulation, and had completed one of two study-related clinical placements. The number of students assessed to be trained, ready and suitable for paid, non-supervised patient-related healthcare work was lower than the actual number available due to the low response rate among third-year students. The authors, who also teach the bachelor program in paramedic science at OsloMet, found that at least 5% of third-year students (n $\approx 25)$ were enrolled in the national response through non-supervised clinical placement in the ambulance service and other paid, pandemic-related work during the first five months of the pandemic. The actual total number of students that participated in patient-related healthcare work would therefore be approximately 60 (39%) of the 155 students enrolled at the bachelor program in paramedic science at OsloMet.

Our results indicate that enrolling paramedic students as part of a national response should depend on the students’ level of knowledge, skills and clinical experience. Implementing study-related clinical placements during a pandemic also seems crucial in order to promote the students’ ability to become trained and ready for paid patient-related healthcare work as part of a national response.

Enrolment of paramedic students as part of the healthcare response seems to be safe when taking some important precautions into account:

- Students not ready for unsupervised patient-related healthcare work should be prioritized for completion of studies so that the training of essential healthcare workers continues.
- Students with theoretical knowledge and simulation-acquired skills in emergency medicine, trauma and disaster management but with no clinical experience should be prioritized for study-related clinical placements. Only selectively, and on an individual basis, should students with no clinical experience be considered for unsupervised patient-related healthcare work as part of the response to a national pandemic or other major disasters.
- Students with theoretical knowledge and simulation-acquired skills in emergency medicine, trauma and disaster management and with clinical experience should be considered for unsupervised clinical work as part of a national response to a pandemic or other major disasters.
- Students without the necessary theoretical and simulation-acquired skills and knowledge should only be enrolled in non-patient-related healthcare work, such as administrative- and logistical tasks for the healthcare services. These services are also valuable in the response to a pandemic.

## Conclusion

The COVID-19 pandemic has placed a strain on the operational resources of the ambulance service. Innovative strategies seeking novel temporary changes in government policies and response plans were required in response to this highly demanding situation. Our overall results show that, during the first five months of the pandemic, no paramedic student tested positive for COVID-19 and that paramedic students’ HRQoL was better than the general population in Norway. Students participated in various patient-related and non-patient-related healthcare work, depending on their knowledge, training and skills. Together these findings support the idea that students were motivated to participate in the pandemic response, despite insufficient supplies of PPE. The results indicate that early mobilization of paramedic students in the workforce during highly demanding situations such as a pandemic is possible, and that the students should be considered a valuable operational resource during such crises.

Due to the lack of literature examining paramedic students’ involvement in healthcare crises, future studies should further examine bachelor program curricula dealing with emergency preparedness with respect to the teaching of both theoretical and clinical skills.

## Data Availability

The datasets generated and/or analyzed during the current study are not publicly available due the risk of identification of individuals when variables are combined. A limited dataset with one or a few variables may be available from the corresponding author on request.

## Appendix 1

Let *M* denote a dummy variable indicating a male respondent; *E*_*prim*_*, E*_*sec*_*, E*_*high*_ indicate highest educational levels corresponding to primary school education, secondary school education, and higher education of more than four years; *A*_*30*_ and *A*_*40*_ age groups 30–39 and 40–49, respectively; and *P* a paramedic student, *P*_*Y1*_, *P*_*Y2*_, and *P*_*Y3*_ paramedic students in years 1, 2, and 3, respectively.

$$\begin{aligned} & EQvalue \sim \alpha + \beta_{m} *M + \beta_{a30} + A_{30} \\ & \quad + \beta_{a40} + A_{40} + \beta_{eduprim} *E_{prim} \\ & \quad + \beta_{edusec } *E_{sec } + \beta_{eduhigh} *E_{high} + \beta_{para} *P + \epsilon \\ \end{aligned}$$

$$\begin{aligned} & EQvalue \sim \alpha + \beta_{m} *M + \beta_{a30} + A_{30} + \beta_{a40} \\ &\quad+ A_{40} + \beta_{eduprim} *E_{prim} + \beta_{edusec } *E_{sec } + \beta_{eduhigh} *E_{high} \\ & \quad + \beta_{paraY1} *P_{Y1} + \beta_{paraY2} *P_{Y2} + \beta_{paraY3} *P_{Y3} + \epsilon \\ \end{aligned}$$

## Abbreviations

AGP: Aerosol generating procedures
AKH: Astrid Karina Harring
COVID-19: Coronavirus disease 2019
CPAP: Continuous positive airway pressure
HRQoL: Health-related quality of life
KH: Kristin Häikiö, first author
OsloMet: Oslo Metropolitan University
OUH: Oslo University Hospital
PPE: Personal protection equipment
SARS-CoV-2: Severe Acute Respiratory Syndrome Coronavirus 2
WHO: World Health Organization
